# Tumour-infiltrating CD8^+^ lymphocytes and colorectal cancer recurrence by tumour and nodal stage

**DOI:** 10.1038/s41416-019-0540-4

**Published:** 2019-08-07

**Authors:** Mark A. Glaire, Enric Domingo, Anita Sveen, Jarle Bruun, Arild Nesbakken, George Nicholson, Marco Novelli, Kay Lawson, Dahmane Oukrif, Wanja Kildal, Havard E. Danielsen, Rachel Kerr, David Kerr, Ian Tomlinson, Ragnhild A. Lothe, David N. Church

**Affiliations:** 1 Cancer Genomics and Immunology Group, The Wellcome Centre for Human Genetics, University of Oxford, Roosevelt Drive, Oxford, OX3 7BN UK; 2 Department of Oncology, University of Oxford, Oxford, UK; 3 Department of Molecular Oncology, Institute for Cancer Research & K.G. Jebsen Colorectal Cancer Research Centre, Oslo University Hospital, Oslo, Norway; 4 Department of Gastroenterological Surgery & K.G. Jebsen Colorectal Cancer Research Centre, Oslo University Hospital, Oslo, Norway; 5 Institute for Clinical Medicine, University of Oslo, Oslo, Norway; 6 Department of Statistics, University of Oxford, Oxford, UK; 7 Department of Histopathology, UCL, Rockefeller Building, University Street, London, WC1E 6JJ UK; 8 Institute for Cancer Genetics and Informatics, Oslo University Hospital, Oslo, Norway; 9 Department of Informatics, University of Oslo, Oslo, Norway; 10 Nuffield Division of Clinical Laboratory Sciences, University of Oxford, Oxford, OX3 9 DU UK; 11 Oxford Cancer Centre, Churchill Hospital, Oxford University Hospitals Foundation NHS Trust, Oxford, UK; 12 Institute of Cancer and Genomic Sciences, University of Birmingham, Edgbaston, Birmingham, B15 2TT UK; 13 Oxford NIHR Comprehensive Biomedical Research Centre, Oxford University Hospitals NHS Foundation Trust, Oxford, UK

**Keywords:** Colorectal cancer, Tumour biomarkers, Tumour immunology

## Abstract

**Background:**

Intratumoural T-cell infiltrate intensity cortes wrelaith clinical outcome in stage II/III colorectal cancer (CRC). We aimed to determine whether this association varies across this heterogeneous group.

**Methods:**

We performed a pooled analysis of 1804 CRCs from the QUASAR2 and VICTOR trials. Intratumoural CD8^+^ and CD3^+^ densities were quantified by immunohistochemistry in tissue microarray (TMA) cores, and their association with clinical outcome analysed by Cox regression. We validated our results using publicly available gene expression data in a pooled analysis of 1375 CRCs from seven independent series.

**Results:**

In QUASAR2, intratumoural CD8^+^ was a stronger predictor of CRC recurrence than CD3^+^ and showed similar discriminative ability to both markers in combination. Pooled multivariable analysis of both trials showed increasing CD8^+^ density was associated with reduced recurrence risk independent of confounders including DNA mismatch repair deficiency, *POLE* mutation and chromosomal instability (multivariable hazard ratio [HR] for each two-fold increase = 0.92, 95%CI = 0.87–0.97, *P* = 3.6 × 10^−3^). This association was not uniform across risk strata defined by tumour and nodal stage: absent in low-risk (pT3,N0) cases (HR = 1.03, 95%CI = 0.87–1.21, *P* = 0.75), modest in intermediate-risk (pT4,N0 or pT1-3,N1-2) cases (HR = 0.92, 95%CI = 0.86–1.0, *P* = 0.046) and strong in high-risk (pT4,N1-2) cases (HR = 0.87, 95%CI = 0.79–0.97, *P* = 9.4 × 10^−3^); *P*_INTERACTION_ = 0.090. Analysis of tumour *CD8A* expression in the independent validation cohort revealed similar variation in prognostic value across risk strata (*P*_INTERACTION_ = 0.048).

**Conclusions:**

The prognostic value of intratumoural CD8^+^ cell infiltration in stage II/III CRC varies across tumour and nodal risk strata.

## Background

Colorectal cancer (CRC) is a substantial cause of morbidity and mortality worldwide. More than half of cases are diagnosed at stage II/III, for which management is typically curative-intent resection followed by adjuvant chemotherapy depending on recurrence risk. Unfortunately, current risk stratification—based on factors such as lymph node involvement, pT4 primary or absence of DNA mismatch repair deficiency (MMR-D)^1,2^—is imprecise, leading to considerable under- and over-treatment.^3,4^ Of the efforts to improve this, perhaps the most promising involves the quantification of the intratumoural T-cell infiltrate, high density of which is associated with improved clinical outcome in CRC.^5–12^ Importantly, in several studies, this relationship has been shown to persist after adjustment for MMR-D.^9–15^ However, these studies have not adjusted for other potential confounders such as chromosomal instability (CIN)—present in more than two thirds of CRCs^16^ and associated with decreased T-cell infiltrate and poor prognosis^17,18^—or *POLE* mutation, which correlates with enhanced immunogenicity and excellent outcome.^19^ In most cases, they have also not addressed the clinically important question of whether the prognostic value of intratumoural T-cell infiltrate in stage II/III CRC is uniform across pT/N stage-based risk strata, or indeed by molecular factors such as *KRAS* and *BRAF* mutation, and finally, they are limited by their use of non-trial, observational series, which are well recognised to suffer a greater risk of bias than meticulously curated clinical trial samples.^20^

In this study, we sought to address these shortcomings by pooled analysis of two large clinical trials and a validation cohort of seven independent series.

## Methods

### Patient selection for biomarker study

Details of the QUASAR2 and VICTOR trials have been reported previously.^21,22^ Briefly, QUASAR2 was an open-label, randomised, controlled trial investigating the addition of bevacizumab to capecitabine in the adjuvant treatment of stage II/III CRC. The final intention to treat population comprised 1941 patients of whom 1715 (88.3%) had colonic tumours and 226 (11.6%) had rectal tumours. The VICTOR trial investigated the efficacy of rofecoxib (a selective cyclooxygenase-2 [COX-2] inhibitor) following completion of standard therapy (surgery ± adjuvant chemotherapy) for stage II–III CRC. The intention to treat analysis included 2434 cases of whom 1592 (65.4%) had colonic tumours and 842 (34.6%) had tumours of the rectum/rectosigmoid junction. Cases from the QUASAR2 and VICTOR trials were identified for inclusion in this biomarker study based on the availability of tumour tissue microarrays (TMAs) and clinical outcome data. Cases treated with preoperative radiotherapy were excluded. All tumours were either stage II or III and had undergone confirmed R0 resection. Data on molecular covariables were not mandated for study inclusion but were available in most cases (see Statistical Analysis). Details of the seven series which formed our pooled validation cohort have either been previously published,^23–27^ or are freely available from the NCBI Gene Expression Omnibus (GEO).^28^ These were all non-experimental datasets, selected for their documentation of primary tumour and nodal stage, *CD8A* expression and clinical outcome.

### Tumour molecular analysis and immunohistochemistry

Tissue microarrays were constructed using punches taken from the centre of the tumour in formalin-fixed paraffin-embedded blocks following identification by the study pathologists; the tumour invasive margin was not sampled. Molecular analyses and immunohistochemistry (IHC) for CD8 and CD3 were performed as reported previously (See Supplementary Methods for full details).^16,19,21,29,30^ Marker positive cells were quantified by computerised analyses using ImmunoPath 1.3.9.0 (Room4, Crowborough, UK), and expressed as the proportion of CD8^+^ or CD3^+^ cells in the total number of cell nuclei across all TMA cores for each case. In addition to the analysis of TMA cores, a subset of 51 cases from the QUASAR2 trial also underwent similar analysis of full face tissue sections to permit comparison between of measurements between the two methods, and comparison of marker densities between the tumour centre and its invasive margin. In view of their similar characteristics,^19^ *POLE*-mutant and MMR-D tumours were combined for all analyses. Molecular analyses in the validation series have been previously reported.^23–26^ Expression of *CD8A*, which encodes the CD8 receptor, was performed by either RNAseq^23^ or expression arrays.^24–26^ Gene expression data were log2 transformed, if not already done, and scaled to permit pooling of series.

### Statistical analysis

Full details of the statistical methods used in this biomarker study are provided in Supplementary Methods. Analyses were performed and reported in accordance with the REMARK guidelines,^31^ and are detailed in Table S1. Survival curves were plotted using the Kaplan–Meier method and compared by the log-rank test. Our primary and secondary objectives were the association of CD8^+^ density, analysed as a continuous variable, with time to CRC recurrence (TTR) (defined as the time from randomisation to CRC relapse, with censoring at last contact or death in case of no recurrence), and overall survival (OS), respectively, in the pooled QUASAR2 and VICTOR cohorts. Exploratory objectives were the association of CD8^+^ cell density with clinical outcome after dichotomisation, and according to tumour and nodal stage, and other clinically relevant risk factors. These objectives were evaluated by pooled univariable and multivariable Cox proportional hazards models, stratified by trial. CD8^+^ cell density was log2 transformed prior to inclusion in regression models (see Supplementary Statistical Methods). Missing covariables data were uncommon (maximum 11.6% missing data for any covariable) and were imputed by multiple imputation by chained equations prior to regression (see Statistical Methods for details). For the final multivariable models, we prespecified the inclusion of variables of clinical importance or known prognostic value and those that demonstrated statistically significant association with CD8^+^ cell density in our prior analysis. The remaining variables were subjected to stepwise backward elimination to remove those which did not contribute to model fit. Details of model diagnostics are provided in the Supplementary Methods. Statistical tests were two-sided, and hypothesis testing was performed at the 5% significance level.

## Results

### QUASAR2 and VICTOR trial patient details

**Fig. 1:**
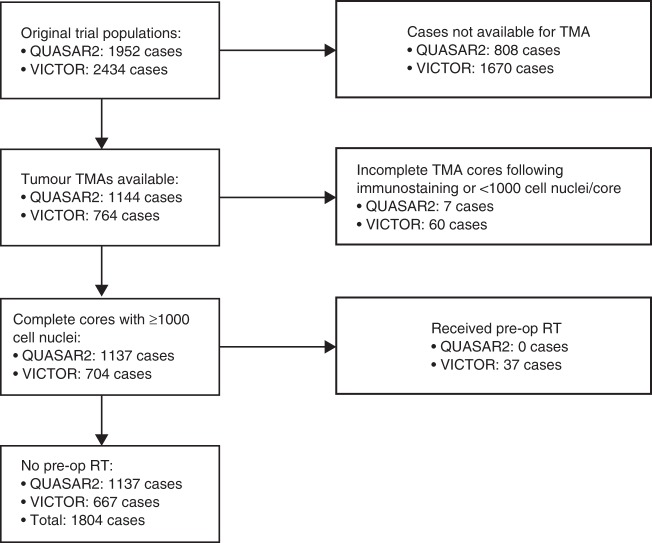
CONSORT diagram for biomarker-evaluable patients. TMA, tissue microarray; Pre-op RT, pre-operative radiotherapy

The CONSORT diagram for this biomarker study is shown in Fig. 1, and the baseline demographic characteristics and clinicopathological and molecular variables of the QUASAR2 and VICTOR cases analysed are presented in Table S2. The cases informative for biomarker analyses were similar to the total trial populations in respect of age, sex, disease stage, use of systemic therapy, disease recurrence and death to the total in both cases (Table S3). Given their similarity, we combined both studies for most subsequent analyses.

### T-cell infiltration in the QUASAR2 study

Analysis of intratumoural TMA cores (taken from the tumour centre) from the QUASAR2 cohort revealed similar numbers of infiltrating CD8^+^ and CD3^+^ cells per mm^2^ to those reported in previous reports^15^ (Table S4), and a highly statistically significant correlation between the densities (defined as the proportion of positive cells to total cell nuclei) of both markers (Spearman rho 0.65; *P* = 2.2 × 10^−16^). Complementary analysis of whole tissue sections in a subset of 51 cases revealed similar positive correlations between estimates of CD8^+^ infiltrate in the tumour centre with those from TMA cores (Spearman rho 0.64; *P* = 3.1 × 10^−5^), and between CD8^+^ cell density in the tumour centre and invasive margin (Spearman rho 0.73, *P* = 1.3 × 10^−9^). In light of these findings, we proceeded to use the results obtained from analysis of TMA cores to examine the association of CD8^+^ and CD3^+^ cell density, alone and in combination, with time to CRC recurrence (TTR) in this trial population, given the known prognostic value of these markers in CRC.^7,14^ This revealed that intratumoural CD8, but not CD3, was significantly associated with TTR in univariable analysis, and in a model that included both markers (Table S4). As the CD8-only model also had similar discrimination ability and smaller Akaike information criterion (AIC—an estimator of model quality, which balances goodness of fit and parsimony), compared with the bivariate model (Table S5), we focused our subsequent analyses on CD8 infiltrate alone.

### CD8^+^ cell density in the combined QUASAR2 and VICTOR cohorts

Unadjusted analyses of the combined trials demonstrated that CD8^+^ cell density was significantly associated with stage II versus III disease, tumour right-sidedness, MMR-D or *POLE* mutation, lack of chromosomal instability (CIN), *BRAF* mutation and absence of disease recurrence and death, but not age, sex, pT stage or *KRAS* mutation (Table S6). Similar associations were evident in the individual trials with the exception of *BRAF* mutation and CIN status, which were not statistically significant in VICTOR perhaps owing to its smaller size.

### CD8^+^ cell density and clinical outcome in the pooled QUASAR2 and VICTOR cohorts

**Fig. 2:**
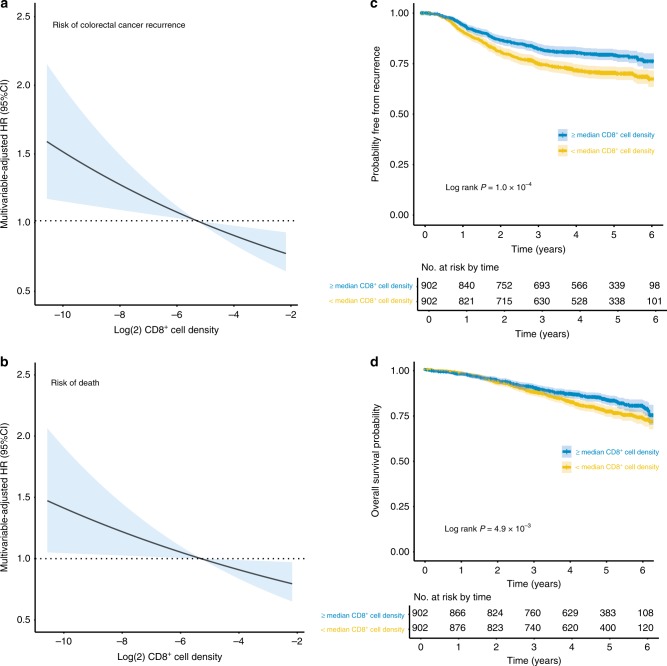
Relationship between tumour CD8^+^ cell density, colorectal cancer recurrence, and overall survival in pooled QUASAR2 and VICTOR studies. Multivariable-adjusted hazard ratios for **a** colorectal cancer recurrence and **b** overall survival according to tumour (log2 transformed) CD8^+^ cell density (i.e. each unit change represents a two-fold increase or decrease in CD8^+^ cell density). Hazard ratio is shown relative to the recurrence risk of tumours with CD8^+^ cell density at the median of the pooled study populations. Corresponding Kaplan–Meier plots for colorectal cancer recurrence (**c**) and overall survival (**d**) for tumours with high and low CD8^+^ cell density using a cut point at the sample median. Shaded area in panels indicates 95% confidence interval (95% CI) for estimate of hazard ratio/survival function. *P* values in **c**, **d** were obtained by the Log-rank test

We next determined the relationship between tumour CD8^+^ density (analysed as a log2 transformed, continuous variable) and CRC recurrence in the pooled trials. Increasing CD8^+^ density was associated with significantly longer TTR in univariable analysis (HR = 0.90 for each two-fold increase; 95% CI = 0.85–0.95, *P* = 1.7 × 10^−4^) (Table 1). While the prognostic factors MMR-D/*POLE* mutation and CIN were also significantly associated with TTR in univariable analyses, only CD8^+^ density remained prognostic in multivariable analyses (HR = 0.92; 95% CI = 0.87–0.97, *P* = 3.6 × 10^−3^) (Table 2, S7, Fig. 2a). Corresponding analysis of overall survival (OS) revealed that CD8^+^ density, but not MMR-D/*POLE* mutation or CIN, was associated with significantly reduced mortality (multivariable-adjusted HR = 0·93; 95% CI = 0·87–0·99, *P* = 0·024) (Table 1, Fig. 2b). For comparison with previous reports, we divided cases into CD8^+^-high and CD8^+^-low categories at the sample median, noting that this resulted in comparable proportions to studies that have used a cut point defined by its relationship with clinical outcome.^8,13^ Multivariable analysis of TTR and OS confirmed better outcome for CD8^+^-high tumours (HR = 0.71, 95% CI = 0.59–0.87, *P* = 7.2 × 10^−4^, and HR = 0.71; 95% CI = 0.57–0.88, *P* = 1.6 × 10^−3^) (Fig. 2c, d). These effect sizes were similar to those reported for a similar recent analysis of 600 cases from the North Central Cancer Treatment Group (NCCTG) N1047 trial^32^

**Table 1:** Univariable and multivariable analyses of time to colorectal cancer recurrence and overall survival in pooled VICTOR and QUASAR2 trial population

	No.	TTR events	OS events	Univariable analysis				Multivariable analysis			
				Time to recurrence		Overall survival		Time to recurrence		Overall survival	
				HR (95% CI)	*P*	HR (95% CI)	*P*	HR (95% CI)	*P*	HR (95% CI)	*P*
Age (continuous)	1804	435	350	1.01 (1.00–1.02)	0.10	1.03 (1.02–1.04)	6.2 × 10^–6^	1.00 (0.99–1.01)	0.43	1.02 (1.01–1.04	1.0 × 10^−4^
Sex											
Male	1083	281	222	1.0 (ref)	–	1.0 (ref)	–	1.0 (ref)	–	1.0 (ref)	–
Female	721	154	128	0.83 (0.68–1.01)	0.063	0.89 (0.72–1.11)	0.31	0.83 (0.68–1.02)	0.075	0.89 (0.71–1.11)	0.30
Location											
Left	972	167	172	1.0 (ref)	–	1.0 (ref)	–	1.0 (ref)	–	1.0 (ref)	–
Right	730	167	158	1.08(0.93–1.31)	0.45	0.77 (0.62–0.96)	0.019	1.05 (0.85–1.31)	0.63	0.83 (0.65–1.06)	0.13
Stage											
II	708	115	91	1.0 (ref)	–	1.0 (ref)	–	1.0 (ref)	–	1.0 (ref)	–
III	1096	320	259	1.89 (1.52–2.40)	6.4 × 10^−9^	1.85 (1.45–2.35)	5.6 × 10^−7^	1.96 (1.57–2.44)	1.34 × 10^−9^	2.03 (1.59–2.60)	1.3 × 10^−8^
Primary tumour											
pT1-3	1239	248	186	1.0 (ref)	–	1.0 (ref)	–	1.0 (ref)	–	1.0 (ref)	–
pT4	552	191	158	1.96 (1.61–2.37)	1.0 × 10^−11^	2.12 (1.70–2.61)	1.1 × 10^−11^	2.14 (1.76–2.60)	3.0 × 10^−14^	2.20 (1.77–2.74)	1.3 × 10^−12^
*BRAF* mutation											
Wild-type	1412	325	250					1.0 (ref)			
Mutant	194	55	52	1.33 (1.00–1.78)	0.048	1.68 (1.25–2.27)	6.5 × 10^−4^	1.59 (1.18 - 2.17)	2.7 × 10^−3^	1.55 (1.12–2.16)	8.0 × 10^−3^
MMR & *POLE* status											
MMR-P & *POLE* wild-type	1412	357	272	1.0 (ref)	–	1.0 (ref)	–	1.0 (ref)	–	1.0 (ref)	–
MMR-D or * POLE*-mutant	230	40	42	0.68 (0.49–0.94)	0.022	1.00 (0.73–1.39)	0.98	0.72 (0.50–1.05)	0.090	0.96 (0.65–1.41)	0.85
Chromosomal instability											
CIN low	550	110	88	1.0 (ref)	–	1.0 (ref)	–	1.0 (ref)	–	1.0 (ref)	–
CIN high	1049	278	214	1.37 (1.10–1.71)	0.0051	1.27 (0.99–1.62)	0.063	1.17 (0.93–1.49)	0.18	1.21 (0.94–1.58)	0.14
Bevacizumab treatment											
No	1222	279	223	1.0 (ref)	–	1.0 (ref)	–	1.0 (ref)	–	1.0 (ref)	–
Yes	582	156	127	1.31 (1.03–1.67)	0.027	1.25 (0.96–1.63)	0.095	1.28 (1.00–1.62)	0.047	1.23 (0.95–1.60)	0.12
CD8^+^ cell density (continuous, log2 transformed)	1804	435	350	0.90 (0.85–0.95)	1.7 × 10^−4^	0.91 (0.86–0.98)	3.2 × 10^−3^	0.92 (0.87–0.97)	3.6 × 10^−3^	0.93 (0.87–0.99)	0.024

Univariable hazard ratios are derived from complete case analyses. Multivariable-adjusted hazard ratios are adjusted for all other covariables listed, and represent estimates derived from ‘final’ Cox models following stepwise backward elimination of candidate variables that did not contribute to model fit using the likelihood ratio test (NB: forced entry variables and variables significantly associated with CD8^+^ cell density were not subjected to variable selection). Results from ‘full’ Cox models including all candidate predictors (age, sex, tumour location, disease stage, primary tumour stage, *BRAF* mutation, *KRAS* mutation, MMR-D/*POLE* mutation, CIN, adjuvant chemotherapy, adjuvant bevacizumab and adjuvant rofecoxib), both before and after the addition of CD8^+^ cell density are provided in Table S5. The addition of CD8^+^ cell density to the model containing all other covariables was associated with an improvement in model fit in both the ‘final’ Cox model above (Akaike Information Criterion [AIC] = 5634.0 vs. AIC 5640.6; Likelihood ratio test for comparison of nested models: *P* = 3.6 × 10^−3^), and the initial, ‘full’ Cox model (Table S3). *TTR* time to colorectal cancer recurrence, *OS* overall survival, *HR* hazard ratio, *95% CI* 95% confidence interval, *pT* pathological tumour (T) stage, *MMR* DNA mismatch repair, *MMR-P* mismatch repair proficient, *MMR-D* mismatch repair deficient, *POLE-mutant* pathogenic *POLE* exonuclease domain mutation

**Table 2:** Colorectal cancer recurrence and overall survival according to tumour risk strata and CD8^+^ cell density in pooled VICTOR and QUASAR2 trial population

	No.	No. events	Predicted proportion event free at 3 years		Univariable analysis			Multivariable analysis		
			25th centile CD8^+^ cell density (95% CI)	75th centile CD8^+^ cell density (95% CI)	HR (95% CI)	*P*	*P*_INTERACTION_	HR (95% CI)	*P*	*P*_INTERACTION_
Time to recurrence										
Low risk (pT3, N0)	453	60	0.90 (0.88 –0.93)	0.90 (0.88–0.93)	1.02 (0.86–1.20)	0.85	0.072	1.03 (0.87–1.21)	0.75	0.090
Intermediate risk (pT4, N0 or pT1-3, N1/2)	1035	242	0.79 (0.76–0.82)	0.82 (0.80–0.85)	0.91 (0.85–0.98)	0.016		0.92 (0.86–1.0)	0.046	
High risk (pT4, N1/2)	303	132	0.58 (0.53–0.64)	0.69 (0.63–0.75)	0.86 (0.78–0.96)	4.7 × 10^−3^		0.87 (0.79–0.97)	9.4 × 10^−3^	
Overall survival										
Low risk (pT3, N0)	453	52	0.95 (0.94–0.97)	0.95 (0.93–0.96)	1.03 (0.87–1.23)	0.72	0.051	1.04 (0.87–1.24)	0.69	0.056
Intermediate risk (pT4, N0 or pT1-3, N1/2)	1035	177	0.89 (0.87–0.91)	0.90 (0.88–0.92)	0.94 (0.86–1.03)	0.20		0.94 (0.86–1.03)	0.22	
High risk (pT4, N1/2)	303	120	0.75 (0.71–0.80)	0.82 (0.78–0.86)	0.88 (0.79–0.97)	0.017		0.88 (0.79–0.98)	0.022	

Point estimates of probability of colorectal cancer recurrence and overall survival are derived from univariable Cox regression of CD8^+^ cell density as a continuous variable (corresponding estimates by the Kaplan–Meier estimator for cases dichotomised at the median CD8^+^ cell density are shown in Table S6). Both point estimates and univariable hazard ratios are derived from complete case analyses. Multivariable-adjusted hazard ratios are adjusted for age, sex, tumour location, *BRAF* mutation, MMR-D/*POLE* mutation, CIN and adjuvant bevacizumab. Tests for interaction are from the cross product term of tumour risk stratum and log2 CD8^+^ cell density in bi-variable and multivariable models

*HR* hazard ratio, *95% CI* 95% confidence interval, *pT* pathological tumour (T) stage

### Prognostic value of intratumoural CD8^+^ cell density by tumour and molecular risk strata

**Fig. 3:**
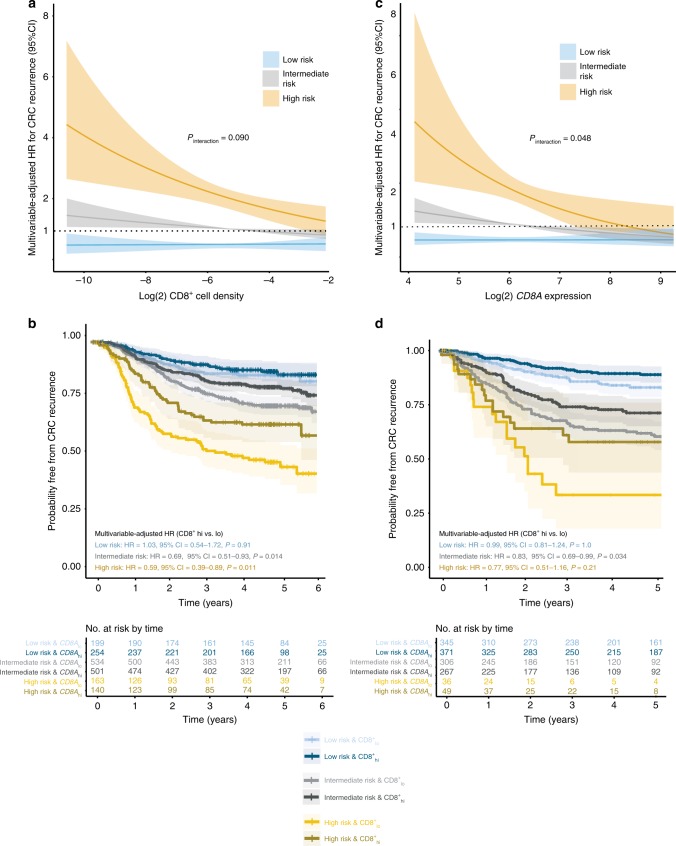
Relationship between tumour CD8^+^ cell density and colorectal cancer recurrence according to primary tumour and nodal status in the pooled QUASAR2 and VICTOR studies, and in the validation cohort. **a** Multivariable-adjusted estimates of the risk of colorectal cancer recurrence in pooled QUASAR2 and VICTOR studies according to tumour (log2 transformed) CD8^+^ cell density for low-risk (pT1-3, N0), intermediate-risk (pT4, N0 and pT1-3, N1/2) and high-risk (pT4, N1/2) tumours (i.e. each unit change represents a two-fold increase or decrease in CD8^+^ cell density). **b** Kaplan–Meier curves showing probability of colorectal cancer recurrence in the QUASAR2 and VICTOR studies for CD8^+^ high and CD8^+^ low tumours (divided at the pooled cohort median) across risk strata. **c** Multivariable-adjusted estimates of the risk of colorectal cancer recurrence according to tumour *CD8A* expression in the pooled validation series for low-risk (pT1-3, N0), intermediate-risk (pT4, N0 and pT1-3, N1/2) and high-risk (pT4, N1/2) tumours. *CD8A* expression values within series were transformed to have mean of zero and unit standard deviation before pooling, and were re-scaled to the mean and standard deviation within the largest series (GSE39582) for ease of interpretation. **d** Kaplan–Meier curves showing probability of colorectal cancer recurrence in the pooled validation series between *CD8A* high and *CD8A* low tumours (divided at the pooled cohort median) across risk strata. *P* value in **a**, **c** indicates results of test for interaction between tumour risk stratum and log2 *CD8A* expression in multivariable Cox model. Shaded areas in **a**, **b**, **c**, **d** indicate 95% confidence intervals

We next explored whether the prognostic value of CD8^+^ cell infiltrate varied by clinically relevant clinicopathological and molecular factors in our trial cohorts. CD8^+^ cell density was significantly associated with recurrence irrespective of patient age (<70 vs. ≥70 years), sex, tumour sidedness, *KRAS* and *BRAF* mutation, and while the correlation was not statistically significant in MMR-D/*POLE*-mutant or CIN-low subgroups, the hazard ratios were similar to those in MMR-P/*POLE*-wild-type and CIN-high subgroups, respectively. In contrast, corresponding univariable and confirmatory multivariable analysis suggested this association (between CD8^+^ cell infiltrate and TTR) differed between pT1-3 vs. pT4 tumours (multivariable-adjusted HR = 0.96, 95% CI = 0.89–1.04, *P* = 0.35 vs. HR = 0.87, 95% CI = 0.80–0.95, *P* = 1.9 × 10^−3^), and to a lesser extent, between node negative and node positive tumours (HR = 0.94, 95% CI = 0.84–1.06, *P* = 0.31; and HR = 0.91, 95% CI = 0.85–0.97, *P* = 5.0 × 10^−3^, respectively). As pT4 primary and node positivity portended similarly reduced TTR in multivariable analysis (Table 1), and pT4,N0 and pT1-3,N1/2 tumours had very similar outcome in the pooled trials (Fig. S1A), we grouped tumours into low (pT1-3, N0), intermediate (pT4, N0 or pT1-3, N1 or N2) and high (pT4, N2) risk groups and examined the correlation of CD8^+^ cell density with TTR across these strata. This suggested an apparent variation in its association, from essentially absent in low-risk cases (HR = 1.03, 95% CI = 0.87–1.21, *P* = 0.75), to modest in intermediate-risk cases (HR = 0.92, 95% CI = 0.86–1.00, *P* = 0.046) and strong in high-risk cases (HR = 0.87, 95% CI = 0.79–0.97, *P* = 9.4 × 10^−3^), although formal testing for an interaction was not statistically significant (*P*_INTERACTION_ = 0.090) (Table 2, Fig. 3a). The apparent discordance translated into even greater variation between strata in the absolute risk of recurrence of tumours with sparse and dense CD8^+^ cell infiltrate. For example, in the low-risk group, 3 year recurrence-free probabilities for tumours with CD8^+^ cell density at the 25th and 75th centiles were similar at 90.2% (95% CI 87.8–92.7%) and 90.1% (95% CI 87.7–92.71%), respectively, while the corresponding proportions in the high-risk group were 58.3% (95% CI = 52.8–64.4%) and 68.6% (95% CI = 63.1–74.6%) (Table 2, Fig. S2). The variation between risk strata was also evident, albeit less obvious, when tumours were classified into CD8^+^ high and low groups based on the sample median, as defined above (Fig. 3b, Table S8). Analysis of overall survival revealed a similar tendency to differences in outcome by CD8^+^ cell density between strata (*P*_INTERACTION_ = 0.056), (Table 2).

### External validation of results

As the analysis across risk strata was not prespecified, we sought to validate our findings in an independent cohort. Although no publicly available datasets have quantified CD8 infiltrate by IHC, we identified five series with gene expression data including *CD8A* (which encodes the CD8 alpha chain) in addition to The Cancer Genome Atlas (TCGA) series and a further set of 264 tumours with *CD8A* expression data. In total, our validation set included 1375 stage II/III tumours with details of tumour and nodal staging, MMR and CIN status and clinical outcome data (Fig. S1B). In keeping with previous studies, tumour *CD8A* expression was significantly associated with TTR in multivariable analysis (HR for each 2-fold increase = 0.86, 95% CI = 0.76–0.97, *P* = 0.018). Interestingly, and concordant with our previous results, the strength of this association varied between low, intermediate and high-risk pT/N strata (HR = 0.99, 95% CI = 0.81–1.24, *P* = 1.0 vs. HR = 0.83, 95% CI = 0.69–0.99, *P* = 0.034 vs. HR = 0.77, 95% CI = 0.51–1.16, *P* = 0.21, respectively; *P*_INTERACTION_ = 0.048) (Fig. 3c, d, Tables S9, S10).

## Discussion

In this analysis of 1804 stage II/III CRCs from two clinical trials with external validation, we have shown that while the association of intratumoural CD8^+^ cell density with recurrence is independent of MMR-D, *POLE* mutation and CIN, it appears to vary by primary tumour and nodal status, from absent or minimal in pT3,N0 disease, to strong in pT4,N1/N2 disease. This variation could not easily be explained by fewer events in the low-risk groups, as the hazard ratios in these approximated unities in both the primary and the validation cohorts. Our study both strengthens the evidence for the prognostic value of tumour CD8^+^ infiltrate in CRC, and suggests that the clinical implementation of this novel marker will require careful consideration. Indeed, among stage pT3, N0 tumours—where MMR status often guides decisions on adjuvant chemotherapy^1^—we found no evidence that CD8^+^ cell density was independently prognostic, suggesting that further work is required to identify immune biomarkers of clinical value in this subgroup. In contrast, patients with pT4,N1/2, cancers with low sparse intratumoural CD8^+^ infiltrate had dismal prognosis, raising the possibility of trials testing intensified or novel adjuvant therapies in this subgroup. Importantly, the prognostic value of CD8^+^ cell density did not vary by other clinically relevant factors, including sidedness, MMR-D, *KRAS* and *BRAF* mutation, suggesting that it may complement these in risk stratification.

Arguably the best-known test for evaluation of the anti-tumour immune response is the “Immunoscore”, developed by Galon et al.^15,33^ While the immune markers and cut points used to define categories have evolved during its development, it currently classifies tumours based on CD8^+^ and CD3^+^ cell density in the tumour centre and its invasive margin. The strength of correlation between these markers, and the superior prognostication provided by CD8^+^ density in our preliminary analysis caused us to focus on this marker alone. Though the different methodologies preclude direct comparison, it is notable that our dichotomised analysis revealed a hazard ratio for recurrence of stage III disease broadly similar to that recently reported for Immunoscore in cases from the NCCTG N0147 trial.^32^ However, avoiding classification of continuous variables has several potential advantages in this setting, as may provide more refined prognostication, and avoids the pitfall of assigning substantially different prognoses to tumours with marker values falling narrowly either side of a cut point.^34^ Interestingly, the variation in the prognostic value of tumour CD8 infiltrate by tumour risk strata was much less obvious when analysed as a dichotomiaed variable.

Our study has limitations. For logistical reasons, our analysis used tumour cores rather than whole sections, and focusesd predominantly on CD8^+^cells, as these were more strongly prognostic than CD3^+^cells in our initial analysis. It will therefore be important to determine the impact of evaluation of a larger area, including the tumour invasive margin, as well as the impact of adding additional immune markers, including and beyond CD3, on prognostication; particularly given the recent results reported for Immunoscore in this setting.^15^ Emerging multispectral platforms are of particular relevance to the latter, though these are not yet ready for clinical implementation. Other priorities will be to define the value of intraepithelial and intrastromal CD8^+^ cell density, which our software did not discriminate between, and the impact of intratumoural heterogeneity on prognosis. Finally, because most patients in these trials were treated with adjuvant cytotoxic chemotherapy, though not oxaliplatin, we were not able to determine whether intratumoural CD8^+^ density predicts recurrence risk in the absence of such treatment, or under oxaliplatin-based therapy.

In summary, in our large study with independent validation, we have confirmed that intratumoural CD8^+^ cell density is independent prognostic factor in stage II/III CRC, and shown that this association appears to vary by tumour and nodal risk strata. Confirmation of this finding, and defining the underlying mechanisms, will be important questions for future studies, as will be the investigation of whether CD8^+^ density as can be used to identify patients who could benefit from adjuvant immunotherapy.

## Supplementary information

Supplemental material

## Data Availability

The datasets pertaining to VICTOR and QUASAR2 used during the current study are available from the corresponding author on reasonable request. The datasets used to validate our findings are available from the GEO database and The Cancer Genome Atlas (TCGA) (https://www.ncbi.nlm.nih.gov/geo/; http://firebrowse.org/).
