# Correction: Tumour-infiltrating CD8^+^ lymphocytes and colorectal cancer recurrence by tumour and nodal stage

**DOI:** 10.1038/s41416-019-0590-7

**Published:** 2019-09-23

**Authors:** Mark A. Glaire, Enric Domingo, Anita Sveen, Jarle Bruun, Arild Nesbakken, George Nicholson, Marco Novelli, Kay Lawson, Dahmane Oukrif, Wanja Kildal, Havard E. Danielsen, Rachel Kerr, David Kerr, Ian Tomlinson, Ragnhild A. Lothe, David N. Church

**Affiliations:** 1 Cancer Genomics and Immunology Group, The Wellcome Centre for Human Genetics, University of Oxford, Roosevelt Drive, Oxford, OX3 7BN UK; 2 Department of Oncology, University of Oxford, Oxford, UK; 3 Department of Molecular Oncology, Institute for Cancer Research & K.G. Jebsen Colorectal Cancer Research Centre, Oslo University Hospital, Oslo, Norway; 4 Department of Gastroenterological Surgery & K.G. Jebsen Colorectal Cancer Research Centre, Oslo University Hospital, Oslo, Norway; 5 Institute for Clinical Medicine, University of Oslo, Oslo, Norway; 6 Department of Statistics, University of Oxford, Oxford, UK; 7 Department of Histopathology, UCL, Rockefeller Building, University Street, London, WC1E 6JJ UK; 8 Institute for Cancer Genetics and Informatics, Oslo University Hospital, Oslo, Norway; 9 Department of Informatics, University of Oslo, Oslo, Norway; 10 Nuffield Division of Clinical Laboratory Sciences, University of Oxford, Oxford, OX3 9 DU UK; 11 Oxford Cancer Centre, Churchill Hospital, Oxford University Hospitals Foundation NHS Trust, Oxford, UK; 12 Institute of Cancer and Genomic Sciences, University of Birmingham, Edgbaston, Birmingham B15 2TT UK; 13 Oxford NIHR Comprehensive Biomedical Research Centre, Oxford University Hospitals NHS Foundation Trust, Oxford, UK

**Keywords:** Colorectal cancer, Tumour biomarkers, Tumour immunology

**Correction to:** *British Journal of Cancer* (2019) **121**, 474–482; 10.1038/s41416-019-0540-4, published online 7 August 2019.

Since the publication of this paper, the authors noticed that the funding information for E.D. was not included. The correct funding information is now shown in the Funding section below.

**Funding:** Funding for this study was provided by: Cancer Research UK (C6199/A10417 and C399/A2291), the European Union Seventh Framework Programme (FP7/207- 2013) grant 258236 collaborative project SYSCOL, European Research Council project EVOCAN, the Oxford Cancer Centre, the Oxford NIHR Comprehensive Biomedical Research Centre (BRC), and core funding to the Wellcome Trust Centre for Human Genetics from the Wellcome Trust (090532/Z/09/Z). M.G. is funded by a Wellcome Trust Clinical Training Fellowship. E.D. is supported by the S:CORT consortium which is funded by a grant from the Medical Research Council and Cancer Research UK. D.N.C. is funded by a Health Foundation/Academy of Medical Sciences Clinician Scientist Fellowship. A.S. is funded by a Norwegian Cancer Society Scientist Fellowship (no. 6824048-2016). R.A.L. is supported by the Research Council of Norway (no. 250993) and the Norwegian Cancer Society (no. 182759-2016). M.N. and D.O. were supported by Cancer Research UK and the National Institute for Health Research through the UCL Experimental Cancer Medicine Centre (to D.O.) and UCL Hospitals Biomedical Research Centre (to M.N.). The views expressed are those of the authors and not necessarily those of the NHS, the NIHR, the Department of Health or the Wellcome Trust. The costs of open access publishing were funded by The Wellcome Trust (090532/Z/09/Z). The study funders had no role in the design, analysis, interpretation, or manuscript preparation for this biomarker study. D.N.C. had full access to all study data and had final responsibility for submission of this report.

