# Supplementary material for: Tumour-infiltrating CD8+ lymphocytes and colorectal cancer recurrence by tumour and nodal stage
Source: Br J Cancer. 2019 Aug 7;121(6):474–82. doi: 10.1038/s41416-019-0540-4 (PMC6738075; doi:10.1038/s41416-019-0540-4)
Supplement: Supplementary file 1 — Supplemental material [file 41416_2019_540_MOESM1_ESM.pdf]

Tumour-infiltrating $CD8^+$ lymphocytes and colorectal cancer recurrence by tumour and nodal stage

Glaire et al.

Appendix

Contents

Supplementary Methods 2

Table S1. Biomarker analyses performed and reported in this study 6

Table S2. Baseline characteristics of VICTOR and QUASAR2 trial cohorts 7

Table S3. Comparison of baseline characteristics of VICTOR and QUASAR2 cases included in biomarker study vs. cases not included 9

Table S4. $CD8^+$ and $CD3^+$ positive cells as counts per mm^2 and as proportion of total cells in TMA cores across the QUASAR2 and VICTOR trial cohorts 10

Table S5. Preliminary analyses of association of $CD8^+$ cell density and $CD3^+$ cell density with colorectal cancer recurrence in the QUASAR2 trial 11

Table S6. Relationship between clinicopathological/molecular characteristics and tumour $CD8^+$ cell density in the combined QUASAR2 and VICTOR trial population 12

Table S7. Effect of addition of $CD8^+$ cell density to 'full' Cox proportional hazards model for colorectal cancer recurrence containing all candidate prognostic variables in the pooled VICTOR and QUASAR2 cohorts 13

Table S8. Estimated probabilities of colorectal cancer recurrence and overall survival according to tumour risk strata and $CD8^+$ cell density dichotomized at sample median in the pooled QUASAR2 and VICTOR trial population 14

Table S9. Colorectal cancer recurrence and overall survival according to tumour risk strata and $CD8^+$ cell density in the pooled external validation cohort 15

Table S10. Estimated probabilities of colorectal cancer recurrence according to tumour risk strata and *CD8A* expression dichotomized at sample median in the pooled external validation cohort 16

Figure S1. Time to colorectal cancer recurrence by primary tumour stage and lymph node status in pooled QUASAR2/VICTOR and external validation cohorts 17

Figure S2 year colorectal cancer recurrence probability by CD8+ density, primary tumour stage and lymph node status in pooled QUASAR2/VICTOR studies 18

Supplementary references 19

Supplementary Methods

Tumour molecular analysis and immunohistochemistry

Tumour DNA was extracted from formalin fixed, paraffin embedded (FFPE) tissue using the DNeasy FFPE Kit (Qiagen, Hilden, Germany). Sanger sequencing was used to detect mutations in *KRAS* (exon 2) and *BRAF* (exon 15) [1-3]. For analysis of *POLE* mutations, in VICTOR cases the entire *POLE* nuclease domain was sequenced, whereas in QUASAR2 sequencing of recurrent mutations in exons 9 (P286R), 13 (V411L), and 14 (S459F) was done by either allele specific PCR or Sanger sequencing. It has previously been shown that there is a high concordance between these two methods [4]. In QUASAR2, microsatellite instability (MSI) status was investigated using all five Bethesda markers (BAT25, BAT26, D2S123, D5346, and D17S250)13 and BAT40, a mononucleotide repeat marker. Tumours with 40% or more unstable markers were classified as being microsatellite unstable [1]. In VICTOR, a panel of four Bethesda markers (BAT25, BAT26, D2S123 and D5S346) was used and tumours classified as MSI if they exhibited two or more unstable markers. Tumours with only one unstable marker were further assessed with the BAT40 marker to determine if MSI was present.[3] To determine the presence of chromosomal instability (CIN) in QUASAR2 tumours, prepared cell monolayers were stained with the Feulgen-Schiff technique. Nuclear DNA content was quantified by the Ploidy Work Station Grabber software, version 1.4.12 (Room4, Crowborough, East Sussex, UK) and a Zeiss Axioplan microscope equipped with a 546 nm green filter and a black and white high-resolution digital camera (Axiocam MRM, Zeiss, Jena, Germany). Aneuploid or tetraploid histograms were classed as positive for CIN and diploid histograms classified as negative[1]. In the VICTOR trial, CIN was determined using automated imaged based cytometry on 40μm scrolls cut from FFPE tumour sections [5]. In view of their modest frequency and similar associations with immune response and favourable prognosis in early-stage CRC [4], *POLE*-mutant and MMR-D tumours were combined for all analyses. Immunohistochemistry (IHC) for CD8 (Leica Biosystems PA0183, mouse clone 4B11, ready-to-use formulation) and CD3 (Leica NCL-L-CD3-565, mouse clone LN10, diluted 1:100) was performed as previously reported [4] on duplicate or triplicate TMA tumour cores. The mean area of individual TMA cores was 1.25mm^2 in the QUASAR2 cases, and 0.97mm^2 in the VICTOR cases. CD8 IHC was also performed on a subset of 51 full-face tumour slides from the QUASAR2 trial. Quantification of marker-positive and -negative cell numbers was done by computerized image

analyses using ImmunoPath 1.3.9.0 (Room4, Crowborough, UK) as previously described [6]. Immune cell density was calculated as the proportion of $CD8^+$ or $CD3^+$ cells in the total number of cell nuclei across all cores for each case, after exclusion of TMA cores containing fewer than 1000 cells, or for which visual inspection (performed in all cases) revealed loss of tumour material following immunostaining. Tumour mutation and MMR-D status in the validation series were determined as previously reported [7-10]. Expression of *CD8A*, which encodes the CD8 receptor, was performed by either RNAseq [7] or expression arrays [8-10]. Gene expression data were log2 transformed if not already done, and scaled within each study to give mean of zero and unit standard deviation to permit pooling of series.

Statistical Methods

Analyses in this biomarker study were performed and reported in accordance with the REMARK guidelines [11]. Demographic, clinicopathological and tumour molecular factors were treated as continuous or categorical variables as appropriate, and compared using the parametric unpaired student's t-test, non-parametric Mann-Whitney test, or Fisher's exact test respectively. Survival curves were plotted using the Kaplan-Meier method and compared by the log-rank test. Biomarker analyses reported in this study are listed in Table S1 in accordance with published guidelines [11]. Our primary and secondary objectives were to assess the association of CD8+ density, analysed as a continuous variable, with time to recurrence (TTR) of CRC (defined as the time from randomization to CRC relapse, with censoring at last contact or death in case of no recurrence), and overall survival (OS) (measured as the time from randomization to death from any cause, with censoring at date of last contact in patients still alive) respectively. Exploratory objectives were the association of CD8+ cell density with clinical outcome according to tumour and nodal stage, and other clinically relevant risk factors. These objectives were evaluated by univariable analysis, and after adjustment for demographic, clinicopathological and molecular confounders, by multivariable analysis using Cox proportional hazards models, stratified by trial. The results of exploratory analyses by competing risks regression according to the method of Fine and Gray [12] did not differ appreciably from those from the Cox models. In view of the strongly positively skewed distribution of $CD8^+$ cell density in the QUASAR2 and VICTOR cohorts (skewness=2.46), and demonstration of non-linearity of response on inspection of Martingale residuals, $CD8^+$ cell density was log(2) transformed prior to inclusion in regression

models; exploratory analysis of models fitted using restricted cubic splines [13] following this transformation demonstrated no significant deviation from linearity. Of the 1804 QUASAR2 and VICTOR cases used for final multivariable analyses, data were missing for covariables of tumour location in 102 cases (5.7%), primary tumour stage in 13 cases (0.7%), *KRAS* mutation in 209 cases (11.6%), *BRAF* mutation in 198 cases (11.0%), MMR-D/*POLE* mutation in 162 cases (9.0%) and CIN in 205 cases (11.4%); logistic regression analyses of these missing predictors using completely observed variables as covariates were consistent with a pattern of missing at random (MAR). Missing covariate data were imputed using multiple imputation by chained equations with predicted mean matching [14], using the MICE package in R [15]. Imputation models included all available covariates, event status, and the Nelson-Aalen estimate of the hazard function [16]. Imputed datasets were pooled for multivariable analysis using the fit.mult.impute command in the Hmisc package in R. A sensitivity analysis of complete cases confirmed that the coefficient for $CD8^+$ cell density was essentially unchanged from that obtained using imputed covariables, although the standard error was greater in keeping with the reduced sample size. For the final multivariable analysis model, we pre-specified the inclusion of variables of clinical importance or known prognostic value (age, sex, disease stage, pT4 primary and MMR-D/*POLE*-mutation), and clinicopathological variables that demonstrated statistically significant association with $CD8^+$ cell density (primary tumour location and *BRAF* mutation). In order to obtain a parsimonious model, the remaining variables (*KRAS* mutation, adjuvant chemotherapy, bevacizumab or rofecoxib) were subjected to stepwise backward elimination to remove those which did not contribute to model fit (adjudged by a statistically significant difference in likelihood ratio statistic between models). Exploratory analyses of the prognostic value of $CD8^+$ cell density by tumour and nodal stage were restricted to those cases for which these variables were available, and in the case of multivariable analyses were adjusted for the same covariables as used in the analysis of the complete cohorts. Exploratory tests for interactions were assessed using the Wald test on the cross product term of $CD8^+$ and the other covariables. We used similar methods for the analysis of the relationship between *CD8A* expression and clinical outcome in the pooled validation series, with the exception that covariables were limited to age, sex, tumour location, pT and nodal stage and MMR-D status. Model discrimination was examined using Harrells C-index, and model choice determined by the Akaike Information Criterion (AIC), and the likelihood ratio test in the case of nested models. Proportionality of hazards in Cox models was confirmed by plotting scaled Schoenfeld

residuals. All statistical analyses were performed R, Version 3.3.1 (https://cran.r-project.org), using packages ‘ggplot2’, ‘mice’, ‘rms’ and ‘Hmisc’. All statistical tests were two-sided. Hypothesis testing was performed at the 5% significance level.

Table S1. Biomarker analyses performed and reported in this study

Analysis	Objective	Objectives	Population	Methods	Reported
Association of tumour CD8$^+$ and CD3$^+$ cell density as continuous variables with CRC recurrence	Preliminary	TTR	Stage II/III CRCs from QUASAR2 trial	Univariable and bivariable HR	Main text, Table S4
Association of tumour CD8$^+$ cell density as a continuous variable with CRC recurrence	Primary	TTR	Stage II/III CRCs from QUASAR2 and VICTOR trials	Log rank test, univariable and multivariable adjusted HR stratified by trial	Main text, Tables 2, S5 Figure 2A
Association of tumour CD8$^+$ cell density as a continuous variable with overall survival	Secondary	OS	Stage II/III CRCs from QUASAR2 and VICTOR trials	Multivariable adjusted HR stratified by trial	Main text, Table 3, Figure 2B
Association of tumour CD8$^+$ cell density dichotomized at sample median	Exploratory	TTR, OS	Stage II/III CRCs from QUASAR2 and VICTOR trials	Log rank test, multivariable adjusted HR stratified by trial	Main text, Figure 2C,D
Association of tumour CD8$^+$ cell density as a continuous variable with CRC recurrence across pT/N risk strata*	Exploratory	TTR, OS	Stage II/III CRCs from QUASAR2 and VICTOR trials	Univariable and multivariable adjusted HR within risk strata, test for interaction	Main text, Figure 3A, Figure S2
Association of tumour CD8$^+$ cell density dichotomized at sample median with CRC recurrence across pT/N risk strata*	Exploratory	TTR	Stage II/III CRCs from QUASAR2 and VICTOR trials	Univariable and multivariable adjusted HR within risk strata, test for interaction	Main text, Figure 3B
Association of tumour *CD8A* expression with CRC recurrence across pT/N risk strata	Validation	TTR	Stage II/III CRCs from pooled GEO and TCGA series	Univariable and multivariable adjusted HR within risk strata, test for interaction	Main text, Figure 3C,D, Table S7

TTR – time to recurrence; OS – overall survival; HR – hazard ratio. *Full multivariable model included age, sex, location, *BRAF* mutation, MMR & *POLE* status, chromosomal instability and Bevacizumab treatment.

Table S2. Baseline characteristics of VICTOR and QUASAR2 trial cohorts

	VICTOR		QUASAR2		Combined		***P*** **(VICTOR vs. QUASAR2)**
	No.	%	No.	%	No.	%	
Total	667	100	1137	100	1804	100	
Age (years)							
Median (range)	64.9 (24.6 – 89.1)		65.0 (21.0 – 85.0)		65.0 (21.0 – 89.1)		0.2[*]
Sex							
Male	426	63.9	657	57.8	1083	60.0	0.12[†]
Female	241	36.1	480	42.2	721	40.0	
Unknown	0	0	0	0	0	0	
Disease stage							
II	314	47.1	394	34.7	708	39.2	2.1x 10^{-7}[†]
III	353	52.9	743	65.3	1096	60.8	
Unknown	0	0.0	0	0	0	0	
Primary tumour stage							
pT1-3	520	78.0	719	63.2	1239	68.7	3.7 x 10^{-13}[†]
pT4	134	20.1	418	36.8	552	30.6	
Unknown	13	1.9	0	0	13	0.7	
Primary tumour location							
Right	284	42.6	446	39.2	730	40.5	0.7[†]
Left	368	55.2	604	53.1	972	53.9	
Unknown	15	2.2	87	7.7	102	5.7	
MMR status[‡]							
MMR-P	492	73.2	932	82.0	1424	78.9	0.81[†]
MMR-D	73	10.9	145	12.8	230	12.1	
Unknown	102	15.3	60	5.3	162	9.0	
POLE status[‡]							
POLE wild-type	477	67.0	1090	95.9	1567	86.7	0.76
POLE-mutant	4	0.6	8	0.7	12	0.7	
Unknown	186	27.9	39	3.4	125	6.9	
Chromosomal instability							
CIN high	370	55.5	679	59.7	1049	58.2	0.3[†]
CIN low	178	26.7	372	32.7	550	30.5	
Unknown	119	17.8	86	7.7	205	11.4	
KRAS							
Wild-type	377	56.5	697	61.3	1074	59.5	0.8[†]
Mutant	186	27.9	335	29.5	521	28.9	
Unknown	119	17.8	105	9.2	209	11.6	
BRAF							
Wild-type	509	76.3	903	79.4	1412	78.3	0.1[†]
Mutant	58	8.7	136	12.0	194	10.8	
Unknown	100	15.0	98	8.6	198	11.0	
Chemotherapy							
No	253	37.9	0	0.0	253	14.0	2.2 x 10^{-16}[†]
Yes	414	62.1	1137	100	1551	86	
Unknown	0	0.0	0	0.0	0	0.0	
Bevacizumab							
No	667	100	555	48.8	1222	67.7	2.2 x 10^{-16}[†]
Yes	0	0	582	51.2	582	32.3	
Unknown	0		0		0		
Rofecoxib							
No	330	49.5	1137	100	1467	81.3	2.2 x 10^{-16}[†]
Yes	337	50.5	0	0	337	18.7	
Unknown	0	0	0	0	0	0	
Disease Recurrence							
No	505	75.7	864	76.0	1369	75.9	0.63[∇]
Yes	162	24.3	273	24.0	435	24.1	
Unknown	0	0	0	0	0	0	
Death							
No	542	81.3	912	80.2	1454	80.6	0.14[∇]
Yes	125	18.7	225	19.8	350	19.4	
Unknown	0	0	0	0			
Probability recurrence-free at 5 years (95% CI)	75.6 (72.3 – 78.9)		74.5 (72.3 – 78.9)		74.9 (72.8 – 77.0)		0.63[∇]
Probability alive at 5 years (95% CI)	81.8 (78.9 – 84.9)		79.0 (76.5 – 81.5)		80.1 (78.1 – 82.1)		0.14[∇]

pT –pathological tumour (T) stage; MMR – DNA mismatch repair; MMR-P – mismatch repair proficient; MMR-D – mismatch repair deficient; *POLE*-mutant – pathogenic *POLE* exonuclease domain mutation; *KRAS*-mutant – *KRAS* mutation in codons 12, 13 or 61; *BRAF*-mutant – *BRAF* mutation at codon 600. *determined by unpaired Student's t-test. †determined by Fisher exact test (in cases which marker status was determined). $^{\nabla}$ determined by log-rank test

Table S3. Comparison of baseline characteristics of VICTOR and QUASAR2 cases included in biomarker study vs. cases not included

	VICTOR (included in study)		VICTOR (not included)		*P* value (included vs. not-included)	QUASAR2 (included in study)		QUASAR2 (not included)		*P* value (included vs. excluded)
	No.	%	N	%		No.	%	No.	%	
Total	667	100	1767	100		1137	100	791	100	
Age (years)										
Median (range)	64.9 (24.6 – 89.1)		65 (25 – 89)		0.92*	65.0 (21.0 – 89.1)		65.0 (23.0 – 88.0)		0.46*
Sex										
Male	426	63.9	1132	64.1	1.0†	657	57.8	442	55.9	0.35†
Female	241	36.1	635	35.9		480	42.2	349	44.1	
Unknown	0	0	0	0		0	0	0	0	
Disease stage										
II	314	47.1	853	48.3	0.30†	394	34.7	150	19.0	0.34†
III	353	52.9	914	51.7		743	65.3	641	81.0	
Unknown	0	0.0	0	0		0	0	0	0	
Chemotherapy										
No	253	37.9	627	35.5	0.54†	0	0.0	0	0	1.0†
Yes	414	62.1	1140	64.5		1137	100	791	100	
Unknown	0	0.0	0	0		0	0.0	0	0.0	
Bevacizumab										
No	667	100	1767	100	1.0†	555	48.8	412	52.1	0.14†
Yes	0	0	0	0		582	51.2	379	47.9	
Unknown	0	0	0	0		0		0	0	
Rofecoxib										
No	330	49.5	875	49.5	0.47†	1137	100	791	100.0	1.0†
Yes	337	50.5	892	50.5		0	0	0	0	
Unknown	0	0	0	0		0	0	0	0	
Disease Recurrence										
No	505	75.7	1313	74.3	0.96†	864	76.0	631	79.8	0.06▿
Yes	162	24.3	454	25.7		273	24.0	160	20.2	
Unknown	0	0	0	0		0	0	0	0	
Death										
No	542	81.3	1415	80.1	0.87†	912	80.2	667	84.3	0.07▿
Yes	125	18.7	352	19.9		225	19.8	124	15.7	
Unknown	0	0				0	0	0	0	

*determined by Mann-Whitney U test. †determined by Fisher exact test. ▿determined by log-rank test.

Table S4. $CD8^+$ and $CD3^+$ positive cells as counts per mm^2 and as proportion of total cells in TMA cores across the QUASAR2 and VICTOR trial cohorts

	**QUASAR2**		**VICTOR**	
**Marker**	Cells/mm^2 ± SD (range)	Density (positive cells/total cells) ± SD (range)	Cells/mm^2 ± SD (range)	Density (positive cells/total cells) ± SD (range)
$CD3^+$ cells	595.6 ± 538.4 (2.4–3652.3)	0.095 ± 0.07 (0.003–0.522)	ND	ND
$CD8^+$ cells	231.6 ± 288.4 (0.8–2485.4)	0.043 ± 0.046 (0.00–0.309)	145.0 ± 188.0 (0.3–1966.2)	0.033 ± 0.035 (0.00–0.384)

Table S5. Preliminary analyses of association of CD8$^+$ cell density and CD3$^+$ cell density with colorectal cancer recurrence in the QUASAR2 trial

Variable	HR (95% CI)	P	C index	AIC
**Model 1. CD8$^+$ cell density only (920 cases, 225 events)**				
CD8$^+$ cell density (log2 transformed)	0.90 (0.83–0.98)	0.011	0.549	2975.5
**Model 2. CD3$^+$ cell density only (920 cases, 225 events)**				
CD3$^+$ cell density (log2 transformed)	0.92 (0.83–1.03)	0.17	0.531	2980.1
**Model 3. CD8$^+$ and CD3$^+$ cell density (920 cases, 225 events)**				
CD8$^+$ cell density (log2 transformed)	0.90 (0.81–0.99)	0.030	0.549	2977.5
CD3$^+$ cell density (log2 transformed)	1.01 (0.88–1.16)	0.88		

Models include all cases with data for both variables. Comparison of Model 1 vs. Model 3: C-index=0.549 vs. 0.549; AIC= 2975.5 vs. 2977.5. HR – hazard ratio; AIC – Akaike Information Criterion.

Table S6. Relationship between clinicopathological/molecular characteristics and tumour CD8$^+$ cell density in the combined QUASAR2 and VICTOR trial population

	No.	Median CD8$^+$ cell fraction, % (IQR)	*P**
Total	1804	2.5 (0.01-38.5)	–
Age (years)			
<65	902	2.6 (1.1–5.2)	0.06
≥65	902	2.4 (1.1–4.8)	
Sex			
Male	1083	2.5 (1.1–4.9)	0.43
Female	721	2.6 (1.1–5.2)	
Disease stage			
II	708	3.0 (1.3–5.4)	1.5×10^{-5}
III	1096	2.2 (1.0–4.7)	
Primary tumour stage			
pT1-3	1239	2.4 (1.1–4.9)	0.15
pT4	552	2.7 (1.2–5.4)	
Unknown	13	2.6 (0.9–3.9_	
Location			
Right	730	3.1 (1.3–6.0)	4.1×10^{-6}
Left	972	2.2 (1.0–4.6)	
Unknown	102	2.5 (0.9–4.8)	
MMR & *POLE* status‡			
MMR-P & *POLE* wild-type	1412	2.4 (1.0–4.7)	4.9×10^{-12}
MMR-D or *POLE*-mutant	230	4.1 (1.9–7.8)	
Unknown	162	2.6 (1.2–6.0)	
Chromosomal instability			
CIN low	550	2.9 (1.3–5.4)	1.2×10^{-3}
CIN high	1049	2.4 (1.0–4.8)	
Unknown	205	2.8 (1.2–6.0)	
KRAS			
Wild-type	1074	2.5 (1.1–4.9)	0.70
Mutant	521	2.5 (1.1–5.0)	
Unknown	209	2.6 (1.2–6.0)	
BRAF			
Wild-type	1412	2.4 (1.1–4.8)	3.2×10^{-3}
Mutant	194	3.1 (1.5–5.9)	
Unknown	198	3.1 (1.2–6.1)	
CRC recurrence			
No	1369	2.7	$7.3 \times 10^{-5\nabla}$
Yes	435	2.0	
Death			
No	1454	2.7	$3.2 \times 10^{-3\nabla}$
Yes	350	2.0	

IQR – interquartile range; pT – pathological tumour stage; MMR – DNA mismatch repair; MMR-P – mismatch repair proficient; MMR-D – mismatch repair deficient; *POLE*-mutant – pathogenic *POLE* exonuclease domain mutation; *KRAS*-mutant – *KRAS* mutation in codons 12, 13 and 61; *BRAF*-mutant – *BRAF* mutation at codon 600. * determined by non-parametric Mann-Whitney U test. $^\nabla$determined by log-rank test

Table S7. Effect of addition of CD8$^+$ cell density to 'full' Cox proportional hazards model for colorectal cancer recurrence containing all candidate prognostic variables in the pooled VICTOR and QUASAR2 cohorts

	Multivariable analysis	
**Variable**	**HR (95% CI)**	***P***
Full model including all candidate variables with exception of CD8$^+$ cell density (1804 cases, 435 events)		
Age (continuous)	1.00 (0.99–1.01)	0.42
Sex (female vs. male)	0.82 (0.67 – 1.00)	0.051
Location (right vs. left)	0.93 (0.75 – 1.14)	0.55
Disease stage (III vs. II)	2.04 (1.61 – 2.60)	5.8 x 10^{-9}
Primary tumour stage (pT4 vs. pT1-3)	2.12 (1.74 – 2.59)	9.7 x 10^{-14}
KRAS mutation (mutant vs. wild-type)	1.20 (0.96 – 1.50)	0.12
BRAF mutation (mutant vs. wild-type)	1.70 (1.20 – 2.40)	2.6 x10^{-3}
MMR & *POLE* mutation (MMR- D/POLE-mutant vs. MMR-P and *POLE* wild-type)	0.70 (0.48 – 1.01)	0.058
Chromosomal instability (CIN-high vs. CIN low)	1.22 (0.97 – 1.54)	0.093
Adjuvant chemotherapy (yes vs. no)	0.90 (0.60 – 1.34)	0.62
Adjuvant bevacizumab (yes vs. no)	1.28 (1.00 – 1.62)	0.047
Adjuvant rofecoxib treatment (yes vs. no)	0.82 (0.60 – 1.11)	0.21
Full model including all candidate variables with addition of CD8$^+$ cell density (1804 cases, 435 events)		
Age (continuous)	1.00 (0.99 - 1.01)	0.49
Sex (female vs. male)	0.82 (0.67 – 1.00)	0.056
Location (right vs. left)	0.96 (0.76 - 1.18)	0.65
Disease stage (III vs. II)	2.01 (1.58 - 2.57)	1.2 x 10^{-9}
Primary tumour stage (pT4 vs. pT1-3)	2.13 (1.75 - 2.61)	6.5 x 10^{-14}
KRAS mutation (mutant vs. wild-type)	1.19 (0.95 – 1.49)	0.13
BRAF mutation (mutant vs. wild-type)	1.69 (1.20 - 2.39)	2.9 x 10^{-3}
MMR & *POLE* mutation (MMR- D/POLE-mutant vs. MMR-P and *POLE* wild-type)	0.74 (0.51 - 1.07)	0.11
Chromosomal instability (CIN-high vs. CIN low)	1.21 (0.96 - 1.53)	0.11
Adjuvant chemotherapy (yes vs. no)	0.88 (0.59 – 1.32)	0.55
Bevacizumab treatment (yes vs. no)	1.28 (1.00- 1.62)	0.048
Adjuvant rofecoxib treatment (yes vs. no)	0.82 (0.60 – 1.12)	0.22
CD8$^+$ cell density (log2 transformed)	0.92 (0.87 - 0.97)	3.7 x 10^{-3}

Estimation of model fit by Akaike information criterion (AIC): full model without CD8$^+$ cell density – AIC= 5639.3; full model including CD8$^+$ cell density – AIC=5633.3. Likelihood ratio test for comparison of full model including CD8$^+$ cell density with model without inclusion of CD8$^+$ cell density: P= 3.7x10^{-3}. HR – hazard ratio; pT – pathological tumour (T) stage; MMR – DNA mismatch repair; MMR-P – mismatch repair proficient; MMR-D – mismatch repair deficient; *POLE*-mutant – pathogenic *POLE* exonuclease domain mutation

Table S8. Estimated probabilities of colorectal cancer recurrence and overall survival according to tumour risk strata and $CD8^+$ cell density dichotomized at sample median in the pooled QUASAR2 and VICTOR trial population

Time point (years)	All cases		Low risk (pT3, N0)		Intermediate risk (pT4, N0 or pT1-3, N1/2)		High risk (pT4, N1/2)	
	$CD8^+$ lo % (95% CI)	$CD8^+$ hi % (95% CI)	$CD8^+$ lo % (95% CI)	$CD8^+$ hi % (95% CI)	$CD8^+$ lo % (95% CI)	$CD8^+$ hi % (95% CI)	$CD8^+$ lo % (95% CI)	$CD8^+$ hi % (95% CI)
**CRC recurrence-free probability**								
**3**	76.5 (73.7–79.4)	84 (81.6–86.6)	86.9 (82.2–91.8)	89.6 (85.8–93.6)	79.0 (75.5–82.7)	85.7 (82.6–89.0)	55.2 (47.9–63.7)	67.4 (59.7–76.1)
**5**	70.1 (67.0–73.4)	79.8 (77.0–82.7)	85.6 (80.0–90.8)	86.9 (82.4–91.5)	71.6 (67.6–75.8)	80.7 (77.1–84.5)	45.7 (38.2–54.6)	63.8 (55.8–72.9)
**Overall survival probability**								
**3**	86.4 (84.0–90.0)	89.9 (88.0–91.9)	95.0 (93.4–96.8)	95.0 (93.2–96.8)	88.4 (86.2–90.7)	90.6 (88.7–92.5)	74.0 (68.5–79.8)	82.6 (78.0–87.5)
**5**	75.8 (72.3–79.5)	81.8 (78.8–84.8)	90.8 (87.8–93.8)	90.6 (87.5–93.7)	78.9 (75.5–82.4)	82.6 (79.7–85.6)	55.9 (48.7–64.1)	69.2 (62.3–76.8)

Point estimates are derived from the Kaplan-Meier estimator of the survival function for each group.

Table S9. Colorectal cancer recurrence and overall survival according to tumour risk strata and CD8$^+$ cell density in the pooled external validation cohort

	No.	No. events	Predicted proportion recurrence-free at 3 years		Univariable analysis			Multivariable analysis		
			25th centile CD8A expression (95% CI)	75th centile CD8A expression (95% CI)	HR (95% CI)	*P*	$P_{INTERACTION}$	HR (95% CI)	*P*	$P_{INTERACTION}$
All cases	1375	303	74.6 (70.2–79.4)	77.9 (73.9–82.2)	0.86 (0.76–0.97)	0.017	0.099	0.86 (0.76–0.97)	0.018	0.048
Low risk (pT3, N0)	716	101	85.5 (82.3–89.7)	86.0 (82.4–89.7)	0.98 (0.80–1.20)	0.82		0.99 (0.81–1.24)	1.0	
Intermediate risk (pT4, N0 or pT1-3, N1/2)	573	167	68.7 (63.4–74.5)	74.7 (70.0–79.6)	0.80 (0.68–0.95)	0.01		0.83 (0.69–0.99)	0.034	
High risk (pT4, N1/2)	85	35	40.7 (30.4 –54.4)	56.8 (47.3–68.3)	0.71 (0.47–1.07)	0.10		0.77 (0.51–1.16)	0.21	

Point estimates of probability of colorectal cancer recurrence are derived from univariable Cox regression of CD8A expression as a continuous variable (corresponding results comparing cases dichotomized at the median CD8$^+$ cell density are shown in Table S7). Multivariable models are adjusted for age, sex, tumour location and, in the case of the total pooled population, primary tumour status (pT4 vs. pT1-3), disease stage (III vs. II), mismatch repair and chromosomal instability status. HR – hazard ratio; pT – pathological tumour (T) stage.

Table S10. Estimated probabilities of colorectal cancer recurrence according to tumour risk strata and *CD8A* expression dichotomized at sample median in the pooled external validation cohort

Time point (years)	All cases		Low risk (pT3, N0)		Intermediate risk (pT4, N0 or pT1-3, N1/2)		High risk (pT4, N1/2)	
	$CD8^+$ lo % (95% CI)	$CD8^+$ hi % (95% CI)	$CD8^+$ lo % (95% CI)	$CD8^+$ hi % (95% CI)	$CD8^+$ lo % (95% CI)	$CD8^+$ hi % (95% CI)	$CD8^+$ lo % (95% CI)	$CD8^+$ hi % (95% CI)
**CRC recurrence-free probability**								
**3**	77.1 (73.8–80.5)	83.0 (80.1–86.2)	87.8 (84.3–91.5)	91.4 (88.3–94.6)	68.8 (63.5–74.6)	75.3 (69.9–81.2)	36.9 (22.3–61.1)	63.0 (49.9–79.4)
**5**	72.8 (69.2–76.5)	80.7 (77.5–84.1)	83.8 (79.6–88.1)	89.3 (85.9–92.9)	63.8 (58.1–70.1)	72.6 (66.9–78.9)	36.9 (22.3–61.1)	60.0 (46.7–77.1)
**Overall survival probability**								
**3**	83.0 (79.0–87.2)	85.2 (81.7–89.0)	86.6 (83.6–89.6)	89.3 (86.6–91.9)	81.7 (78.4–85.1)	84.1 (81.1–87.3)	75.3 (67.7–83.7)	76.8 (69.9–84.5)
**5**	73.0 (68.0–78.4)	76.3 (71.2–81.2)	74.4 (69.8–79.4)	79.3 (75.0–83.8)	66.1 (61.3–71.2)	70.2 (65.7–75.0)	55.9 (45.3–69.0)	58.3 (48.3–70.4)

Point estimates are derived from the Kaplan-Meier estimator of the survival function for each group.

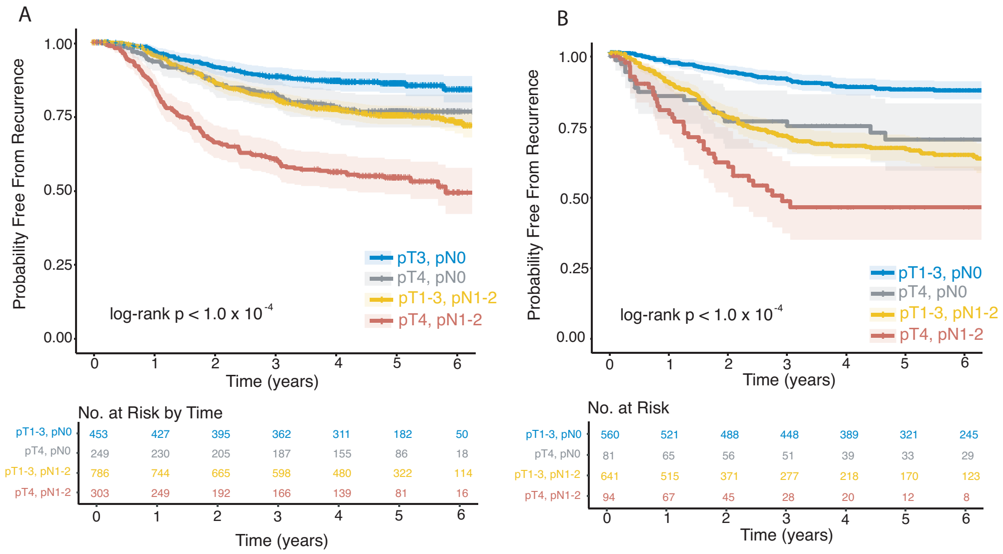

Figure S1. Time to colorectal cancer recurrence by primary tumour stage and lymph node status in pooled QUASAR2/VICTOR and external validation cohorts
Kaplan Meier curves showing time to colorectal cancer recurrence by primary tumour stage (pT1-3 vs. pT4) and lymph node status (N0 vs. N1/2) in stage II/III CRC from the QUASAR2 and VICTOR studies (A) and the pooled validation cohorts (B). Shaded areas represent 95% confidence intervals (95% CIs). Log rank *P* values are for comparison of all groups.

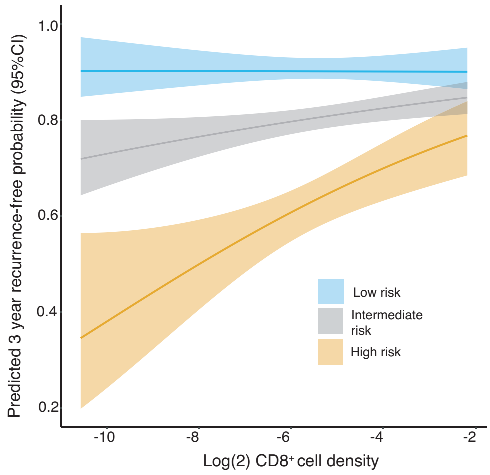

Figure S2 year colorectal cancer recurrence probability by CD8+ density, primary tumour stage and lymph node status in pooled QUASAR2/VICTOR studies

Predicted 3 year recurrence-free probability according to tumour CD8^{+} density by tumour risk strata.

Supplementary references

1. Kerr RS, Love S, Segelov E, Johnstone E, Falcon B, Hewett P et al. Adjuvant capecitabine plus bevacizumab versus capecitabine alone in patients with colorectal cancer (QUASAR 2): an open-label, randomised phase 3 trial. Lancet Oncol 2016; 17(11): 1543-1557; e-pub ahead of print 2016/09/24; doi 10.1016/S1470-2045(16)30172-3.
2. Domingo E, Church DN, Sieber O, Ramamoorthy R, Yanagisawa Y, Johnstone E et al. Evaluation of PIK3CA mutation as a predictor of benefit from nonsteroidal anti-inflammatory drug therapy in colorectal cancer. Journal of clinical oncology : official journal of the American Society of Clinical Oncology 2013; 31(34): 4297-4305; doi 10.1200/JCO.2013.50.0322.
3. Domingo E, Ramamoorthy R, Oukrif D, Rosmarin D, Presz M, Wang H et al. Use of multivariate analysis to suggest a new molecular classification of colorectal cancer. J Pathol 2013; 229(3): 441-448; doi 10.1002/path.4139.
4. Domingo E, Freeman-Mills L, Rayner E, Glaire M, Briggs S, Vermeulen L et al. Somatic POLE proofreading domain mutation, immune response, and prognosis in colorectal cancer: a retrospective, pooled biomarker study. The lancet Gastroenterology & hepatology 2016; 1(3): 207-216; e-pub ahead of print 2017/04/14; doi 10.1016/s2468-1253(16)30014-0.
5. Mouradov D, Domingo E, Gibbs P, Jorissen RN, Li S, Soo PY et al. Survival in stage II/III colorectal cancer is independently predicted by chromosomal and microsatellite instability, but not by specific driver mutations. Am J Gastroenterol 2013; 108(11): 1785-1793; e-pub ahead of print 2013/09/17; doi 10.1038/ajg.2013.292.
6. Blaker YN, Brodtkorb M, Maddison J, Hveem TS, Nesheim JA, Mohn HM et al. Computerized image analysis of the Ki-67 proliferation index in mantle cell lymphoma. Histopathology 2015; 67(1): 62-69; e-pub ahead of print 2014/11/29; doi 10.1111/his.12624.
7. Network TCGA. Comprehensive molecular characterization of human colon and rectal cancer. Nature 2012; 487(7407): 330-337; doi 10.1038/nature11252.
8. Staub E, Groene J, Heinze M, Mennerich D, Roepcke S, Klaman I et al. An expression module of WIPF1-coexpressed genes identifies patients with favorable prognosis in three tumor types. Journal of molecular medicine (Berlin, Germany) 2009; 87(6): 633-644; e-pub ahead of print 2009/04/29; doi 10.1007/s00109-009-0467-y.
9. Marisa L, de Reynies A, Duval A, Selves J, Gaub MP, Vescovo L et al. Gene expression classification of colon cancer into molecular subtypes: characterization, validation, and prognostic value. PLoS medicine 2013; 10(5): e1001453; e-pub ahead of print 2013/05/24; doi 10.1371/journal.pmed.1001453.
10. Sheffer M, Bacolod MD, Zuk O, Giardina SF, Pincas H, Barany F et al. Association of survival and disease progression with chromosomal instability: a genomic exploration of colorectal cancer. Proc Natl Acad Sci U S A 2009; 106(17): 7131-7136; e-pub ahead of print 2009/04/11; doi 10.1073/pnas.0902232106.
11. McShane LM, Altman DG, Sauerbrei W, Taube SE, Gion M, Clark GM. REporting recommendations for tumour MARKer prognostic studies (REMARK). In: Br J Cancer, Vol 932005, pp 387-391.
12. Fine J, Gray, R.A. A proportional hazards model for the subdistribution of a competing risk. J Am Stat Assoc 1999; 94: 496-509.
13. Harrell FE. Regression Modeling Strategies: With Applications to Linear Models, Logistic Regression, and Survival Analysis. Springer: New York, NY, 2001.
14. Marshall A, Altman DG, Holder RL. Comparison of imputation methods for handling missing covariate data when fitting a Cox proportional hazards model: a resampling study. BMC Med Res Methodol 2010; 10: 112; e-pub ahead of print 2011/01/05; doi 10.1186/1471-2288-10-112.
15. van Buuren S, Boshuizen HC, Knook DL. Multiple imputation of missing blood pressure covariates in survival analysis. Stat Med 1999; 18(6): 681-694; e-pub ahead of print 1999/04/16.
16. White IR, Royston P, Wood AM. Multiple imputation using chained equations: Issues and guidance for practice. Stat Med 2011; 30(4): 377-399; e-pub ahead of print 2011/01/13; doi 10.1002/sim.4067.
